# Association between statin use and immune-related adverse events in patients treated with immune checkpoint inhibitors: analysis of the FAERS database

**DOI:** 10.3389/fimmu.2024.1439231

**Published:** 2024-10-08

**Authors:** Huaju Yang, Rendong Huang, Ping Zhang, Yingtong Liu, Zheran Liu, Jiagang He, Xingchen Peng

**Affiliations:** ^1^ Department of Radiation Oncology, Cancer Center, West China Hospital, Sichuan University, Chengdu, Sichuan, China; ^2^ Hangzhou Linan Guorui Health Industry Investment Co., Ltd, Hangzhou, Zhejiang, China; ^3^ Department of Oncology, Chengdu Integrated TCM & Western Medicine Hospital, Chengdu First People’s Hospital, Chengdu, Sichuan, China; ^4^ Department of Biotherapy, Cancer Center, West China Hospital, Sichuan University, Chengdu, Sichuan, China; ^5^ Department of Medical Education, Kweichow Moutai Hospital, Zunyi, Guizhou, China

**Keywords:** immune checkpoint inhibitors (ICIs), statins, immune-related adverse events(irAEs), cancer, food and drug administration adverse event reporting system database (FAERS)

## Abstract

**Background:**

Understanding the risk relationship between statin use and immune-related adverse events (irAEs) in patients undergoing immune checkpoint inhibitors (ICIs) therapy is crucial for optimizing oncological management.

**Objective:**

This study aimed to investigate whether the use of statins increases the risk of irAEs in patients receiving ICI therapy.

**Methods:**

This study primarily utilized data from FAERS database. Multivariable logistic regression was the principal method of analysis, and the Benjamini-Hochberg procedure was employed to adjust for multiple hypothesis testing.

**Results:**

In a group of 145,214 patients undergoing ICI therapy, 9,339 reported using statin medications. Multivariable analysis indicated an increased risk of irAEs among statin users (OR 1.199, 95% CI: 1.141-1.261; FDR p < 0.001) in comparison to those not using statins. Notably, increased risks were observed particularly in patients diagnosed with lung, pancreatic, and renal cancers. The link between statin usage and increased irAEs risk remained consistent across various ICIs treatments.

**Conclusions:**

Statin medication usage is linked to an elevated probability of experiencing irAEs in patients enrolled in ICI therapy. In cancer patients receiving immune checkpoint inhibitors, careful consideration of statin use is essential to avoid potentially increased irAEs risk. These findings provide critical guidance for clinicians in developing treatment strategies that balance therapeutic efficacy and safety in oncological management.

## 1 Introduction

Recent advancements in oncology have been characterized by the introduction of immune checkpoint inhibitors (ICIs) that targeting Cytotoxic T-Lymphocyte Antigen 4 (CTLA-4), Programmed Death-1 (PD-1) and Programmed Death-Ligand 1 (PD-L1) (1). These therapies have transformed the management of various malignancies such as melanoma (2, 3), non-small cell lung carcinoma (NSCLC) (4, 5), and kidney cancer (6) by enhancing T-cell-mediated tumor immunity. The expanding indications for these therapies reflect both increasing clinical experience and a broadening therapeutic impact (7, 8).

Nevertheless, the systemic stimulation of the immune system through ICIs might trigger immune-related adverse events (irAEs) that affect a range of organs, including the skin, gastrointestinal system, hepatic tissue, endocrine systems, and respiratory tract (9, 10). These events range from mild rashes and fatigue to severe, potentially life-threatening conditions such as hepatitis, colitis, and myocarditis (11). irAEs can manifest within days of initiating treatment or as late as one-year post-treatment. The median onset time for irAEs typically spans from 2 to 16 weeks following the commencement of therapy (12). The incidence of irAEs ranges from 54% to 76% (12), signifying that more than half of the patients treated with ICIs encounter at least one adverse reaction. The pathophysiological mechanisms underlying irAEs, though influenced by factors such as genetic predisposition, microbiome composition, and the use of concurrent medications, remain incompletely understood (13, 14). Effective management of irAEs is essential to maintain therapeutic efficacy and preserve the quality of life for patients.

In the complex clinical landscape, about 47% of cancer patients present with comorbid conditions, particularly cardiovascular diseases (15, 16). Notably, the prevalence of hyperlipidemia—a critical risk factor for atherosclerosis—is high among elderly cancer patients. Both the malignancy itself and the treatments for cancer can disrupt lipid metabolism, thereby increasing cardiovascular risk (17). While studies have investigated the potential of statins to enhance the efficacy of ICIs, research into their relationship with the risk of irAEs remains scant. This gap underscores the need for comprehensive studies to explore how statins might modulate the immune response in the context of ICI therapy.

To this end, we utilize records from the Food and Drug Administration (FDA) Adverse Event Reporting System (FAERS) to examine the link between statin use and irAEs among patients undergoing therapy with ICIs. The FAERS database, which aggregates post-marketing adverse drug reaction data from healthcare providers, patients, and manufacturers, serves as a valuable source of real-world evidence for understanding drug safety (18). Our systematic evaluation of FAERS data aims to clarify the relationship between statins and irAEs, thereby facilitating the development of personalized treatment strategies.

## 2 Methods

### 2.1 Data repositories

This study utilized information from the FAERS database, an open-access resource managed by the FDA. The database compiles detailed records of negative reactions and medication mishaps. FAERS is a critical component of the FDA’s system for monitoring the safety of pharmaceuticals and biological treatments after they reach the market. The specific dataset used for our analysis is available at https://fis.fda.gov/sense/app/95239e26-e0be-42d9-a960-9a5f7f1c25ee/sheet/7a47a261-d58b-4203-a8aa-6d3021737452/state/analysis. Access to this data supports transparency and enhances research efforts in pharmacovigilance.

### 2.2 Data acquisition and filtering

**Figure 1:**
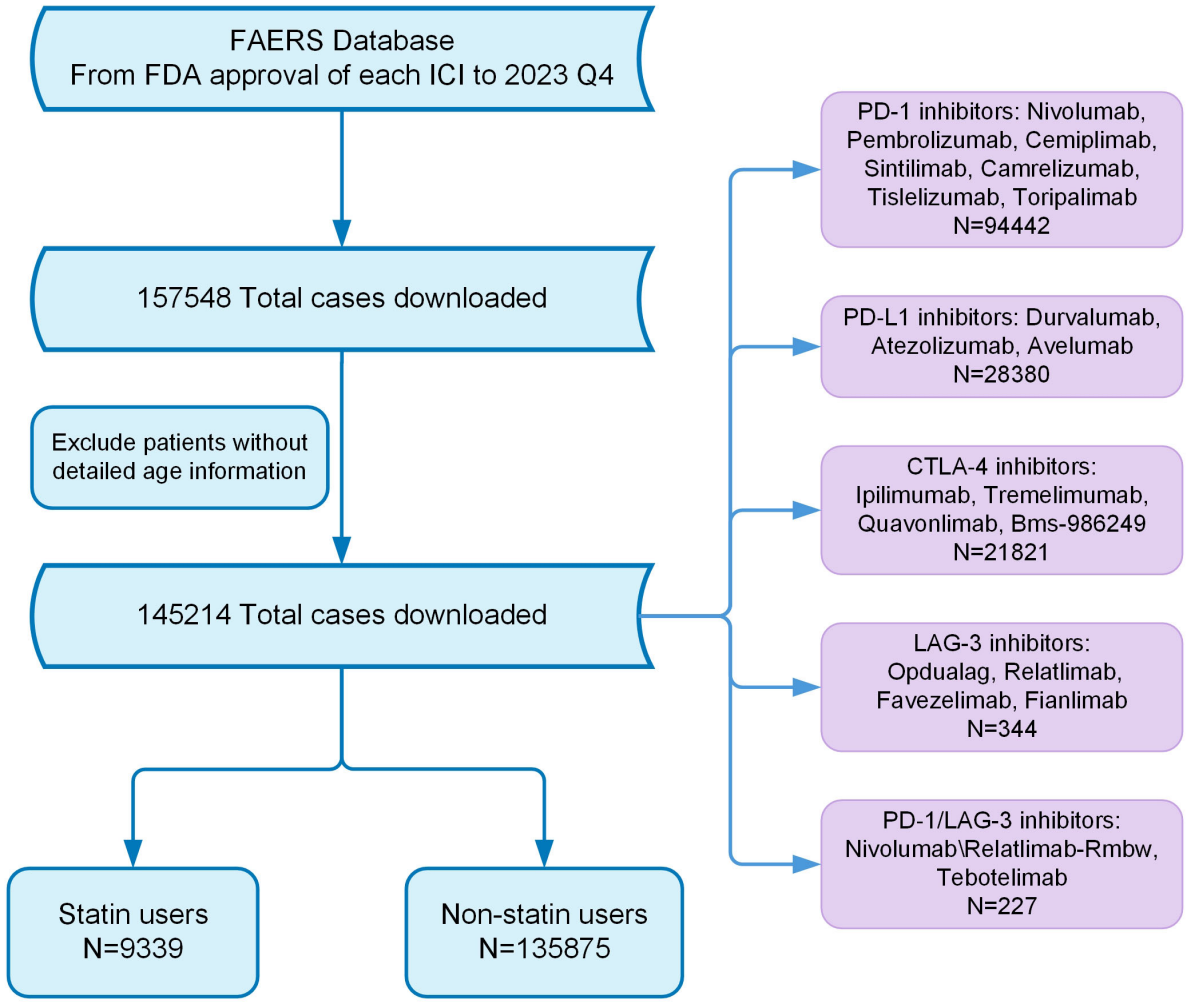
Flow chart showing the analysis process of the study. FDA, Food and Drug Administration; ICI, Immune checkpoint inhibitor.

In this retrospective analysis, we retrieved records of adverse events (AEs) concerning ICIs from the FAERS database. The dataset includes records starting from the FDA approval date of each drug through December 30, 2023. The ICIs examined in this study comprise PD-1 inhibitors such as Nivolumab and Pembrolizumab, PD-L1 inhibitors like Durvalumab and Atezolizumab, CTLA-4 inhibitors including Ipilimumab, Lymphocyte Activation Gene-3 (LAG-3) inhibitors such as Relatlimab, and bispecific PD-1/LAG-3 inhibitors like Nivolumab/Relatlimab-Rmbw. Treatment regimens were categorized based on their composition, including monotherapy and various combinations such as dual immunotherapy and chemotherapy with immunotherapy. Patients were categorized as statin recipients if they used any statin medications during treatment, such as atorvastatin, simvastatin, rosuvastatin, lovastatin, pravastatin, or other statins like fluvastatin and pitavastatin. irAEs were identified according to terminology from peer-reviewed guidelines (19), with affected patients classified into the irAE group. The classification of AEs was based on the primary system organ classes outlined in the Medical Dictionary for Regulatory Activities (20). The detailed study process is outlined in **Figure 1**.

### 2.3 Statistical analysis

To evaluate the link between statin usage and irAEs, multivariable logistic regression was utilized, incorporating adjustments for potential confounders such as age, gender, specific ICIs type, and therapeutic modalities. To control for the risk of false discoveries in multiple comparisons, the Benjamini-Hochberg method was applied using the “p.adjust” function from R’s “stats” package. The analyses were executed using two-tailed tests, with a threshold for statistical significance set at a false discovery rate (FDR) adjusted p-value (FDR p) under 0.05. Information processing and analysis were carried out using R software, version 4.3.3. Analysis stratification included tumor type, adverse reaction type, and organ-specific irAEs to deeply explore the nuanced effects of various statin medications and different classes of ICIs. This layered approach facilitated a robust examination of the interactions between statins and ICIs, enhancing our understanding of their potentially varied effects on patient outcomes within oncological settings.

## 3 Results

### 3.1 Initial patient characteristics

In a comprehensive cohort comprising 145,214 patients treated with ICIs, 9,339 individuals (6.4%) reported using statins at baseline, as illustrated in **Table 1**. Within this subgroup, the distribution was predominantly male (69.3%) compared to female (30.4%), with 0.3% of cases unspecified. The median age for statin users was higher at 69.2 years, compared to 64.0 years for non-users, suggesting that statin users were generally older. Age stratification revealed that 30.7% of statin users were aged between 18 and 65 years, 46.1% between 65 and 75 years, and 23.2% were over 75 years. In contrast, non-users presented with 49.4% in the 18-65 age group, 33.2% in the 65-75 age group, and 17.4% in the over 75 age group. The most common treatment strategy across the entire population was the use of PD-1 antagonists, followed by PD-L1 blockade agents and CTLA-4 blocking antibodies. The largest volume of adverse event reports originated from the United States and Japan, indicating a higher reporting rate or possibly a greater use of these therapies in these nations, as shown in **Table 1**.

**Table 1:** Baseline feature.

Characteristics		With Statin	Without Statin	P value
		n=9339	n=135875	
**Sex**	Female	2838 (30.4%)	51067 (37.6%)	<.001
	Male	6473 (69.3%)	82148 (60.5%)	
	Not Specified	28 (0.3%)	2660 (2%)	
**Age(SD)**		69.2 (8.6)	64.0 (12.6)	<.001
**Age Group**	18-65 years	2870 (30.7%)	67088 (49.4%)	<.001
	65-75 years	4306 (46.1%)	45173 (33.2%)	
	>75 years	2163 (23.2%)	23614 (17.4%)	
**Treatment strategy**	PD1 inhibitor	5313 (56.9%)	89129 (65.6%)	<.001
	PD-L1 inhibitor	2195 (23.5%)	26185 (19.3%)	
	CTLA-4 inhibitor	1764 (18.9%)	20057 (14.8%)	
	LAG-3 inhibitor	48 (0.5%)	296 (0.2%)	
	PD-1/LAG-3 inhibitor	19 (0.2%)	208 (0.2%)	
**Country**	United States	3260 (34.9%)	34013 (25%)	<.001
	Japan	666 (7.1%)	31103 (22.9%)	
	France	853 (9.1%)	14270 (10.5%)	
	Not Specified	687 (7.4%)	10015 (7.4%)	
	Germany	716 (7.7%)	7465 (5.5%)	
	Canada	681 (7.3%)	4112 (3%)	
	China	13 (0.1%)	4307 (3.2%)	
	United Kingdom	493 (5.3%)	3217 (2.4%)	
	Italy	178 (1.9%)	3519 (2.6%)	
	Spain	357 (3.8%)	2577 (1.9%)	
	Other Country	1435 (15.4%)	21277 (15.7%)	
**irAEs**	Yes	2235 (23.9%)	28154 (20.7%)	<.001
	No	7104 (76.1%)	107721 (79.3%)	

### 3.2 Association between statin usage and irAEs in oncology patients undergoing ICI therapy

**Figure 2:**
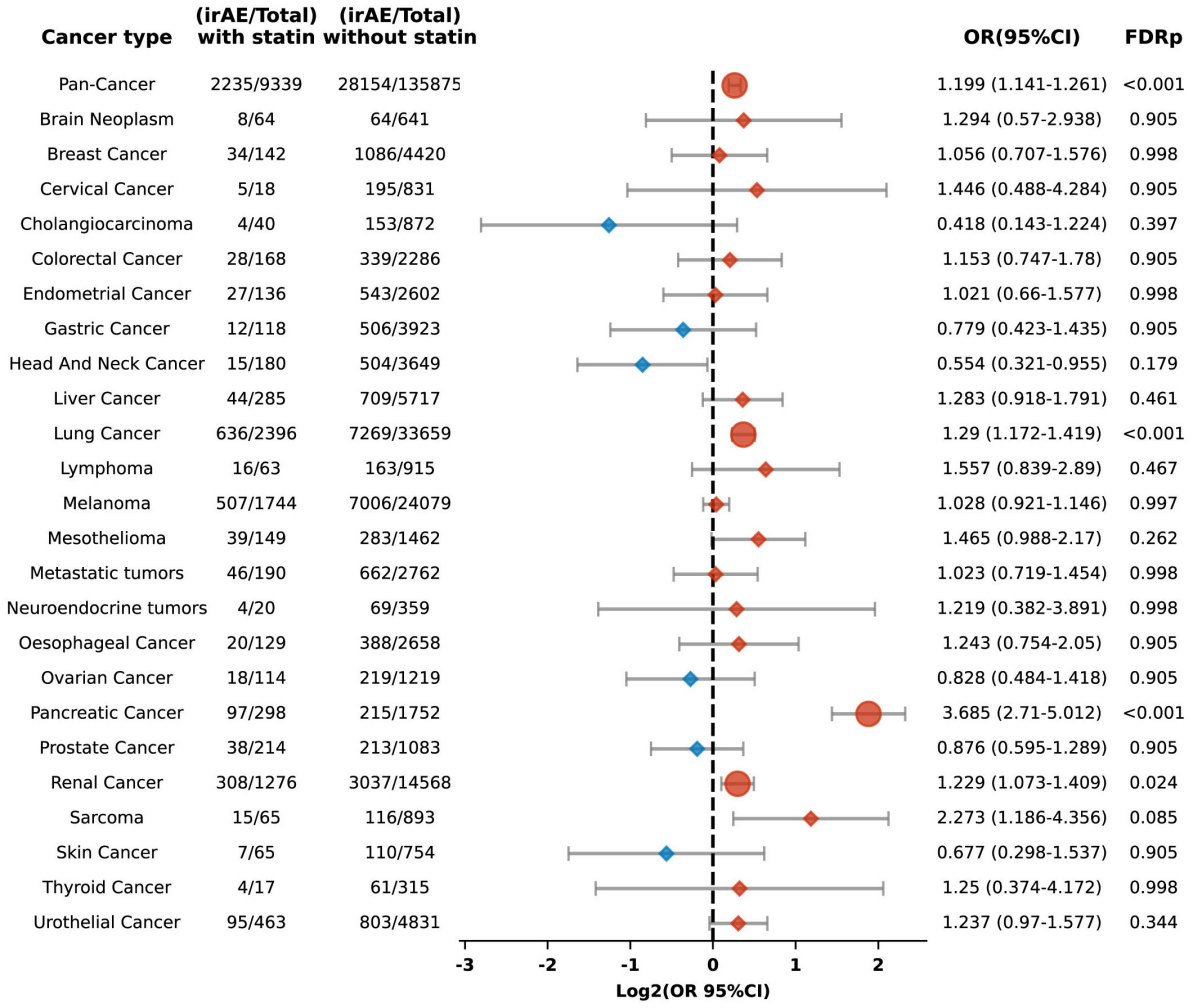
Forest plot illustrating the relationship between statin use and irAEs across various cancer types in patients undergoing ICIs. Red signifies elevated risk among individuals treated with statins, whereas blue denotes increased risk among those not treated with statins. Circles indicate an FDRp < 0.05, suggesting statistically significant findings; diamonds indicate an FDRp ≥ 0.05, indicating findings that are not statistically significant.

In this retrospective cohort study, we evaluated the correlation between statin administration and irAEs within patients treated with ICIs. After controlling for confounders, statin users demonstrated a significantly increased risk of irAEs, with an adjusted odds ratio (OR) of 1.199 (95% confidence interval [CI], 1.141 to 1.261) and an FDR p of less than 0.001. Our study population included patients from 24 distinct cancer types, each represented by a minimum of 200 cases. Stratified analyses by cancer type indicated elevated risks of irAEs in individuals with lung, pancreatic, and renal cancers, with respective ORs of 1.29 (95% CI: 1.172-1.419; FDR p < 0.001), 3.685 (95% CI: 2.71-5.012; FDR p < 0.001), and 1.229 (95% CI: 1.073-1.409; FDR p = 0.024) (**Figure 2**).

Our cohort included 36,055 lung cancer patients, representing one-fourth of the study population. To determine whether the significant increase in irAEs observed in the overall cohort was driven disproportionately by the large subset of lung cancer patients, we conducted an additional multivariate logistic regression analysis excluding those with lung cancer. Even with their exclusion, the link between statin use and a heightened risk of irAEs remained statistically significant, with an OR of 1.183 (95% CI: 1.115-1.255; FDR p < 0.001). This suggests that the observed increase in irAEs is not solely attributable to the lung cancer subgroup but is consistent across the broader population.

**Figure 3:**
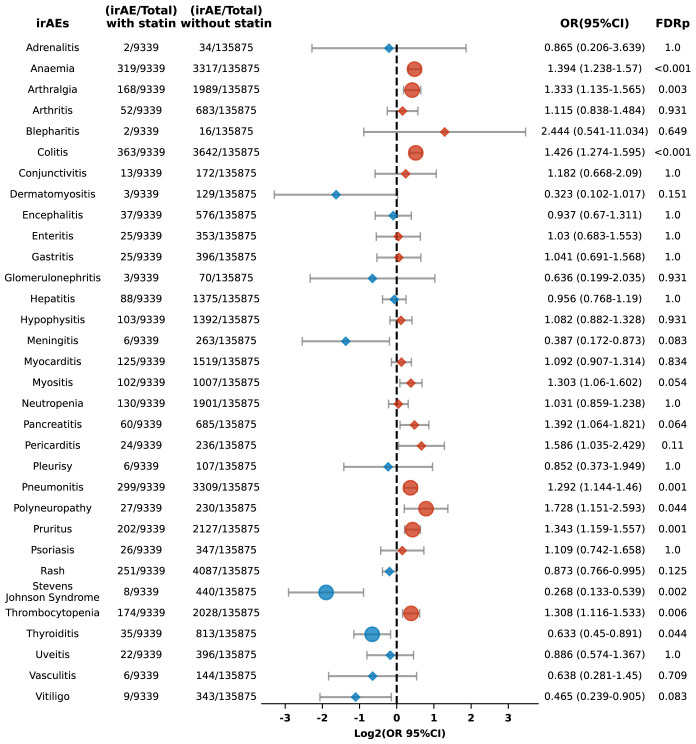
Forest plot illustrating the connection between statin use and different irAEs in patients undergoing ICIs. The icons and color coding utilized in this figure adhere to the same conventions established in **Figure 1**.

Additionally, we performed a sub-analysis focusing on specific irAEs, revealing increased risks of anemia (OR 1.394, 95% CI: 1.238-1.570; FDR p < 0.001), arthralgia (OR 1.333, 95% CI: 1.135-1.565; FDR p = 0.003), colitis (OR 1.426, 95% CI: 1.274-1.595; FDR p < 0.001), pneumonitis (OR 1.292, 95% CI: 1.144-1.460; FDR p = 0.001), polyneuropathy (OR 1.728, 95% CI: 1.151-2.593; FDR p = 0.044), pruritus (OR 1.343, 95% CI: 1.159-1.557; FDR p = 0.001), and thrombocytopenia (OR 1.308, 95% CI: 1.116-1.533; FDR p = 0.006) among statin users compared to non-users (**Figure 3**). Conversely, the risks for Stevens-Johnson syndrome and thyroiditis were lower in statin users, with ORs of 0.268 (95% CI: 0.133-0.539; FDR p = 0.002) and 0.633 (95% CI: 0.45-0.891; FDR p = 0.044), respectively (**Figure 3**).

**Figure 4:**
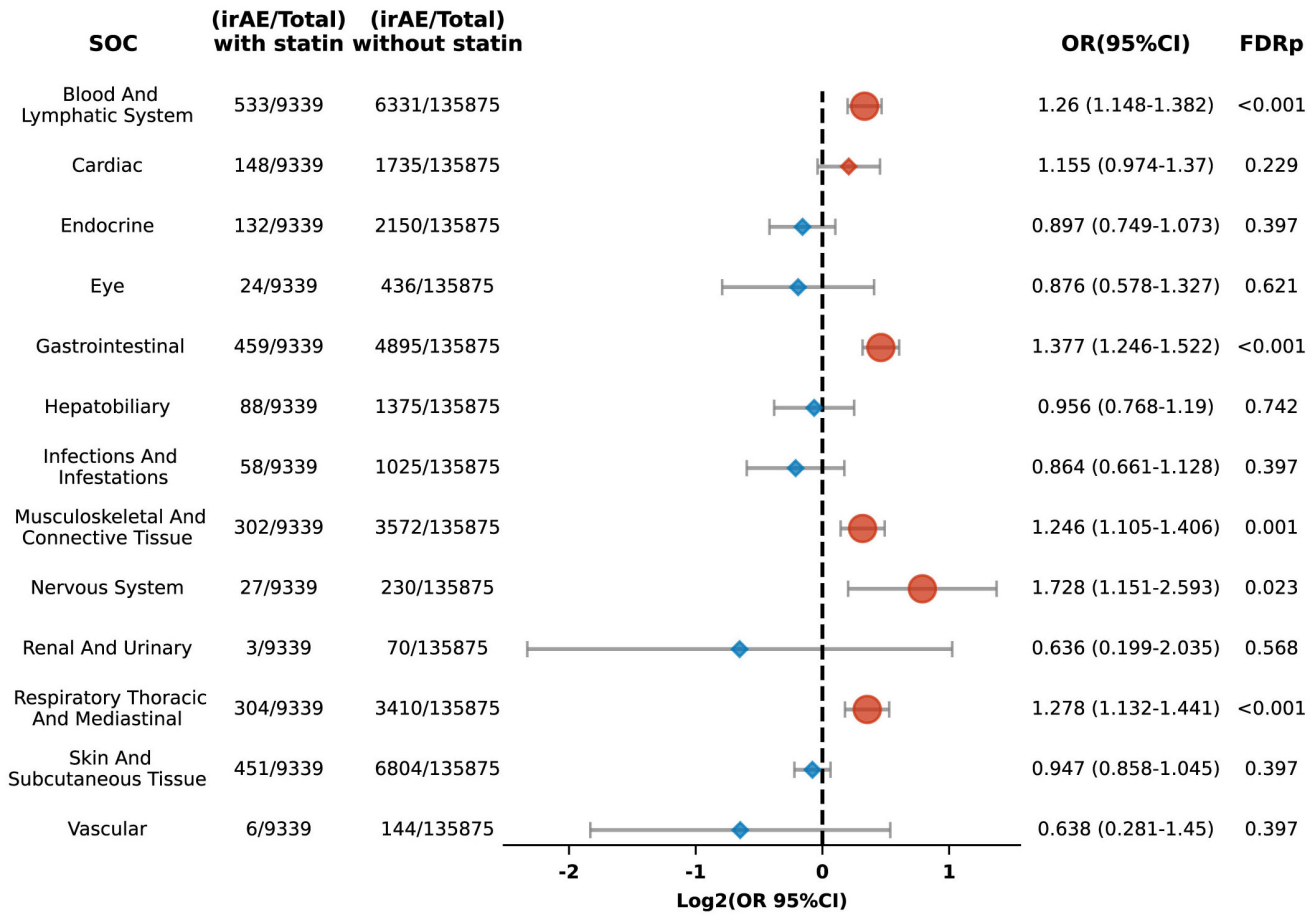
Forest plot illustrating the link between statin use and organ-specific irAEs in patients undergoing ICIs. The icons and color coding utilized in this figure adhere to the same conventions established in **Figure 1**. SOC, system organ classes.

We categorized irAEs into 13 Systemic organ groups and conducted subgroup analyses for organ-specific irAE risks. Among patients treated with ICIs and using statins, significant increases in the risk of irAEs were observed for several systems: the blood and lymphatic system (OR 1.26, 95% CI: 1.148-1.382; FDR p < 0.001), gastrointestinal system (OR 1.377, 95% CI: 1.246-1.522; FDR p < 0.001), musculoskeletal and connective tissue (OR 1.246, 95% CI: 1.105-1.406; FDR p = 0.001), nervous system (OR 1.728, 95% CI: 1.151-2.593; FDR p = 0.023), and the respiratory thoracic and mediastinal systems (OR 1.278, 95% CI: 1.132-1.441; FDR p < 0.001) (**Figure 4**).

### 3.3 Association of different statins with irAEs

**Figure 5:**
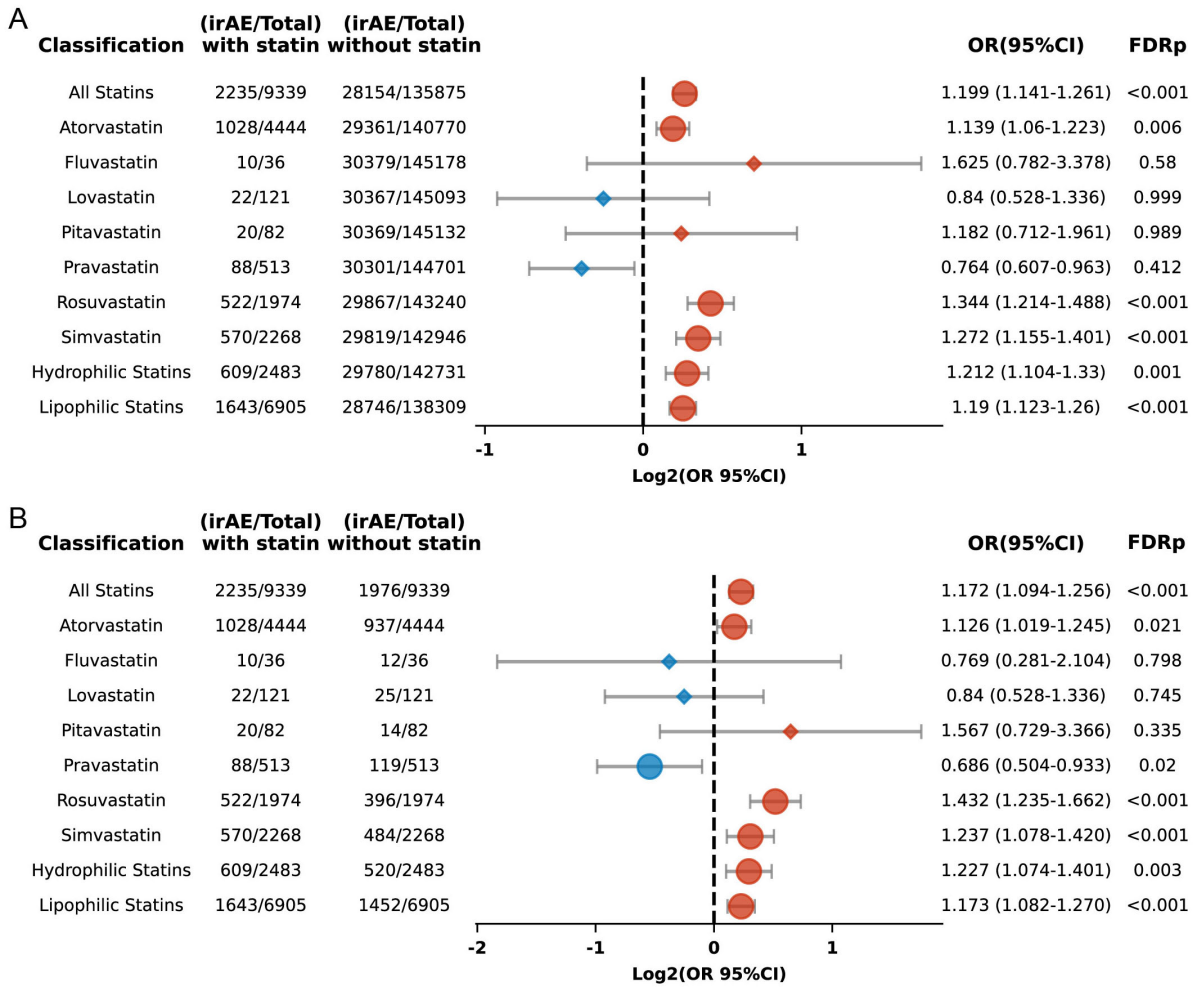
**(A)** Forest plot depicting the association between individual statins and irAEs in ICI-treated patients. **(B)** Forest plot depicting the association between individual statins and irAEs in ICI-treated patients after propensity score matching. The icons and color coding utilized in this figure adhere to the same conventions established in **Figure 1**.

The seven commonly used statin medications were included in this study, which aimed to evaluate the connection between individual statins and the risk of irAEs in the broader population. We found that atorvastatin (OR 1.139, 95% CI: 1.06 to 1.223; FDR p = 0.006), rosuvastatin (OR 1.344, 95% CI: 1.214 to 1.488; FDR p < 0.001), and simvastatin (OR 1.272, 95% CI: 1.155 to 1.401; FDR p < 0.001) were linked with an augmented risk of irAEs (**Figure 5A**). In contrast, fluvastatin, lovastatin, pitavastatin, and pravastatin did not show significant associations. Further analysis using propensity score matching confirmed these associations for six of the statins but revealed that pravastatin showed a diminished risk of irAEs (FDR p = 0.02) (**Figure 5B**).

Rosuvastatin and pravastatin were categorized as hydrophilic statins, whereas the other five statins were classified as lipophilic. Their associations with irAEs were analyzed separately. The results indicated that both lipophilic (OR 1.19; 95% CI: 1.123 to 1.26; FDR p < 0.001) and hydrophilic statins (OR 1.212; 95% CI: 1.104 to 1.33; FDR p = 0.001) (**Figure 5A**) were linked to an increased risk of irAEs, with significant differences maintained even after applying propensity score matching (**Figure 5B**).

### 3.4 Association between statin use and irAEs with different ICIs regimens

Previous studies have suggested variability in the toxicity profiles of different ICIs, but these differences are not yet fully elucidated (1, 21). Our purpose was to assess the association between statin consumption and irAEs across various ICIs treatment regimens. The treatment approaches in our study encompassed antagonists targeting PD-1, PD-L1, CTLA-4, LAG-3, and the combination of PD-1/LAG-3. Due to the limited sample sizes for LAG-3 blockade and PD-1/LAG-3 blockade therapies, we were unable to perform subgroup analyses for these groups. The associations between statin use and irAEs in patients receiving PD-1 blockers, PD-L1 blockers, and CTLA-4 blockers are as follows.

#### 3.4.1 Association of statin use with irAEs in PD-1 inhibitor therapy

**Figure 6:**
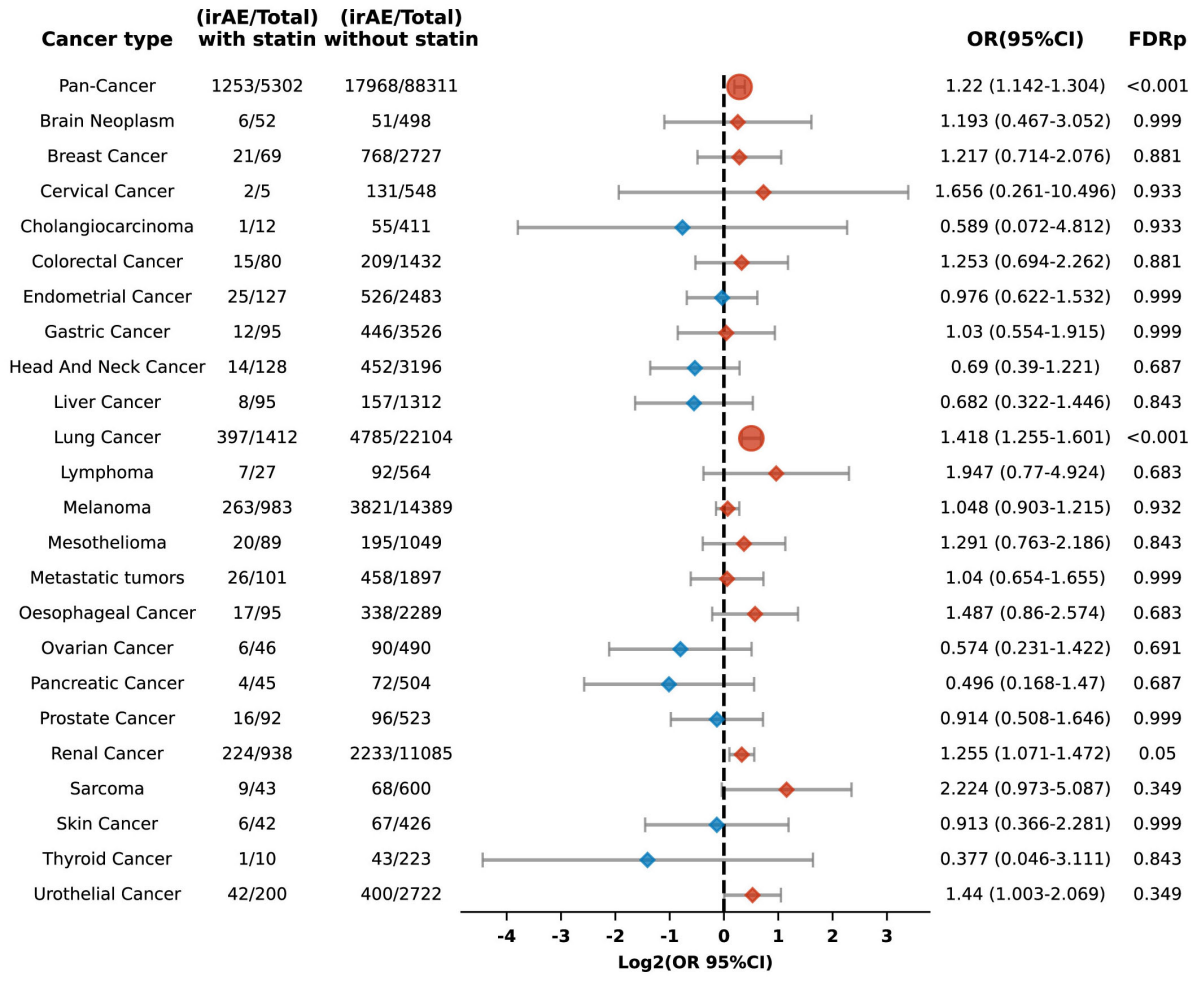
Forest plot illustrating the relationship between statin use and irAEs across various cancer types in patients undergoing PD-1 blockade drugs. The icons and color coding utilized in this figure adhere to the same conventions established in **Figure 1**.

Among 93,613 patients who had been prescribed PD-1 blockade therapy, 5,302 had a history of using statin medications. Our study found that the overall risk of irAEs was higher in statin users relative to non-users (OR 1.22, 95% CI: 1.142-1.304; FDR p < 0.001). Subgroup analysis revealed more pronounced risks for patients with lung cancer (OR 1.418, 95% CI: 1.255-1.601; FDR p < 0.001) (**Figure 6**).

**Figure 7:**
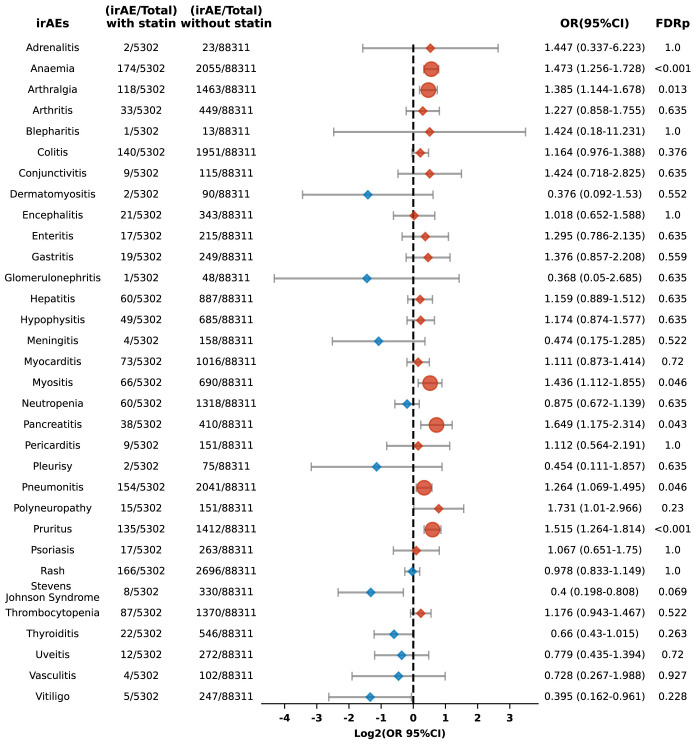
Forest plot illustrating the connection between statin use and different irAEs among patients undergoing PD-1 blockade drugs. The icons and color coding utilized in this figure adhere to the same conventions established in **Figure 1**.

Moreover, the analysis revealed varying increased risks for different types of adverse reactions in statin users, including anemia (OR 1.473, 95% CI: 1.256-1.728; FDR p < 0.001), arthralgia (OR 1.385, 95% CI: 1.144-1.678; FDR p = 0.013), myositis (OR 1.436, 95% CI: 1.112-1.855; FDR p = 0.046), pancreatitis (OR 1.649, 95% CI: 1.175-2.314; FDR p = 0.043), pneumonitis (OR 1.264, 95% CI: 1.069-1.495; FDR p = 0.046), and pruritus (OR 1.515, 95% CI: 1.264-1.814; FDR p < 0.001) (**Figure 7**).

#### 3.4.2 Association of statin use with irAEs in PD-L1 inhibitor therapy

**Figure 8:**
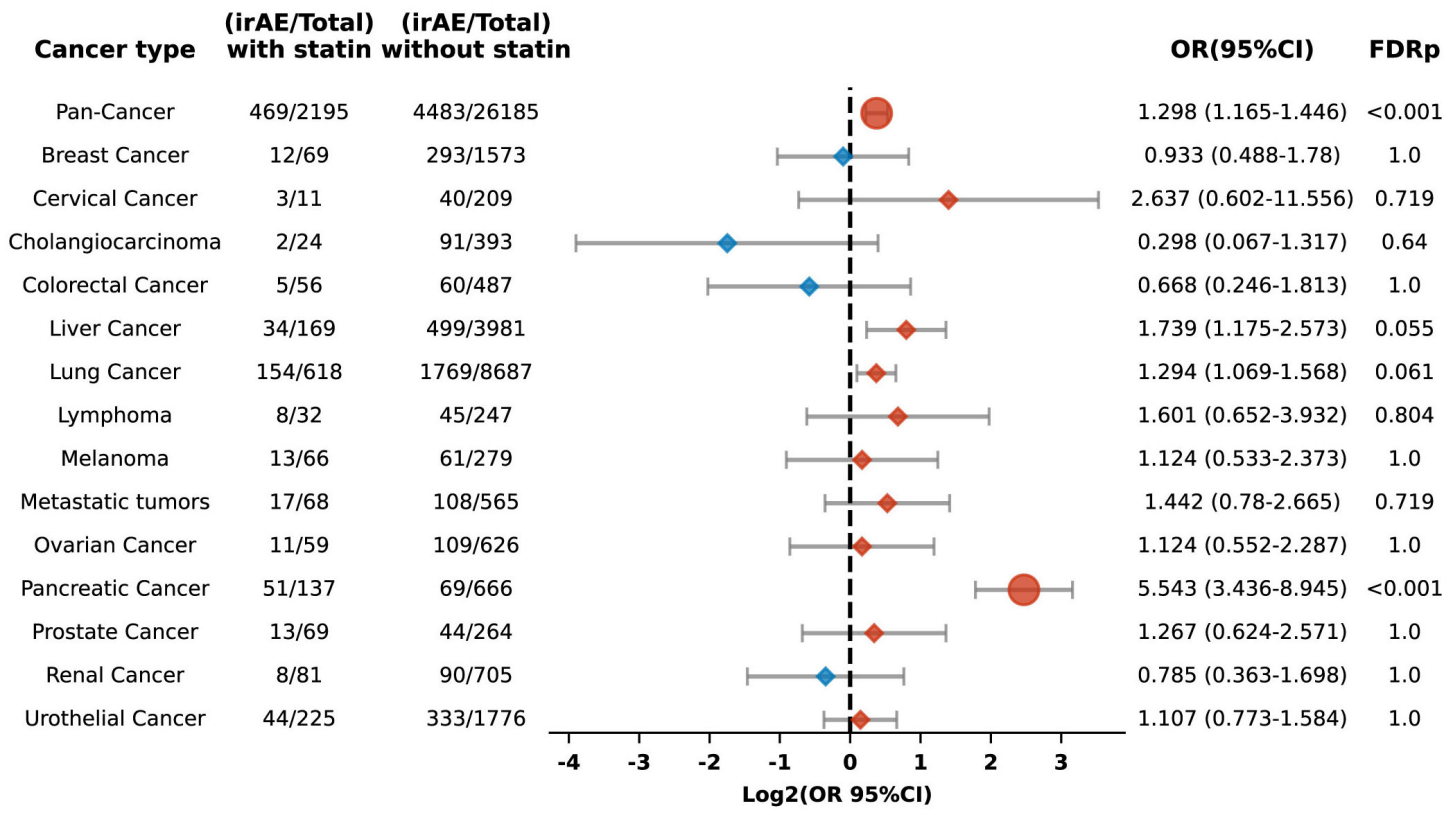
Forest plot illustrating the relationship between statin use and irAEs across various cancer types in patients undergoing PD-L1 blockade drugs. The icons and color coding utilized in this figure adhere to the same conventions established in **Figure 1**.

Among 28,380 patients prescribed PD-L1 blockade therapy, including 2,195 identified as statin users, the study showed that compared to non-users, those with statin use exhibited an increased risk of irAEs (OR 1.298, 95% CI: 1.165-1.446; FDR p < 0.001). This association was particularly pronounced in pancreatic carcinoma, where a significantly elevated risk was observed (OR 5.543, 95% CI: 3.436-8.945; FDR p < 0.001) (**Figure 8**).

**Figure 9:**
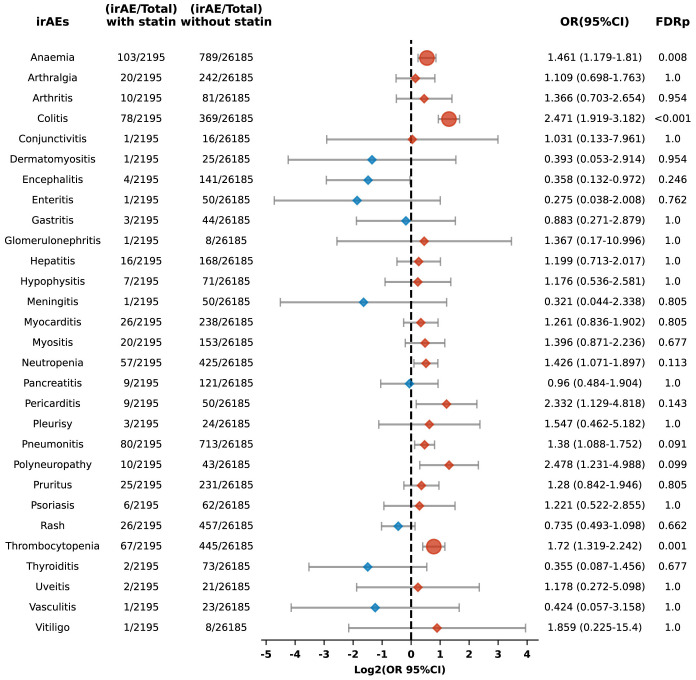
Forest plot illustrating the connection between statin use and different irAEs among patients undergoing PD-L1 blockade drugs. The icons and color coding utilized in this figure adhere to the same conventions established in **Figure 1**.

Additionally, the risk of specific adverse reactions, including anemia, colitis, and thrombocytopenia, was also higher among statin users, with ORs of 1.461 (95% CI: 1.179-1.81; FDR p = 0.008), 2.471 (95% CI: 1.919-3.182; FDR p < 0.001), and 1.72 (95% CI: 1.319-2.242; FDR p = 0.001), respectively (**Figure 9**).

#### 3.4.3 Association of statin use with irAEs in CTLA-4 inhibitor therapy

In the cohort treated with CTLA-4 inhibitors, an analysis revealed a nonsignificant trend toward an enhanced risk of irAEs among patients using statins compared to their non-statin-using counterparts. Nevertheless, a notable exception was identified in the subgroup of patients with pancreatic cancer, where statin use was significantly associated with a greater risk of irAEs (OR 4.867, 95%CI: 2.918-8.117; FDR p<0.001), as detailed in **Supplementary Figure 1**. Across various irAEs, no other significant differences were detected (**Supplementary Figure 2**).

## 5 Discussion

ICIs have emerged as a pivotal component of contemporary oncological therapeutics, but their use can trigger irAEs, affecting multiple organs and potentially limiting the efficacy of ICIs. Given that cancer and cardiovascular diseases often coexist, patients undergoing ICI therapy may also require statins to manage cardiovascular health. However, the relationship between statin use and the development of irAEs remains unclear. Further investigation into this potential association is essential for optimizing the safety and efficacy of ICIs in clinical practice.

In our analysis using the FAERS database, we observed that patients prescribed statins during ICI therapy exhibited a heightened risk of irAEs compared to those not prescribed statins. This inclination has been noted by previous researchers as well. Drobni et al. (22), observed that among patients prescribed statins, 45.3% (389 out of 858 patients) experienced irAEs, compared to 41.8% (789 out of 1889 patients) among those not prescribed statins. However, this difference was not significant statistically (P = 0.087).

Several mechanisms have been suggested to explain the relationship between the statin treatment and the heightened risk of irAEs. Statins work by inhibiting the enzyme HMG-CoA reductase, thereby reducing endogenous cholesterol synthesis. This inhibition leads to a compensatory increase in the expression of low-density lipoprotein receptors in the liver, thereby enhancing the clearance of lipoproteins and achieving a lipid-lowering effect (23). Zhou W. et al. (24) demonstrate that in cancer cells with specific genetic mutations (e.g., ARID1A), statins modulate the mevalonate pathway to induce inflammasome formation, subsequently triggering pyroptosis. This process releases immunostimulatory molecules like IL-1β, enhancing T cell infiltration and activity within the tumor microenvironment. Additionally, research by Xia et al. (25) has revealed that statins block the HMG-CoA reductase enzyme, which is a key component of the mevalonate pathway, thereby impacting the downstream production of geranylgeranyl diphosphate (GGPP). This affects the pre-translational modification of proteins in antigen-presenting cells, specifically inhibiting the geranylgeranylation of small GTPases such as Rab5. This inhibition disrupts the maturation of endocytic bodies within cells, extending the retention time of antigens and enhancing their presentation as well as T-cell activation. These findings underscore the dual utility of statins not only as cholesterol-lowering drugs but also as adjuvants in cancer immunotherapy. Research by Cantini and others further confirms the feasibility of using statins as adjuvants in clinical settings (26).

On the other hand, Ma et al. (27) demonstrated that high cholesterol levels in the tumor microenvironment significantly promote CD8+ T-cell exhaustion by upregulating regulatory receptors like PD-1, thereby weakening immune functionality. Furthermore, Zhou W. et al. (24) found that simvastatin, a cholesterol-lowering agent, reduces intratumoral cholesterol, alleviates CD8+ T-cell exhaustion, and restores or enhances T-cell functionality. Another study’s findings suggest that statins mediate the suppression of PD-L1 in lung cancer and melanoma cells by involving the phosphorylation and activation of β-catenin-S552 via AKT-S473 (28). Moreover, it has been proposed by researchers that statins modulate the inflamed tumor microenvironment (TME) and potentiate anti–PD-1 immunotherapy in NSCLC by transcriptionally repressing PD-L1 and promoting ferroptosis (29).

**Figure 10:**
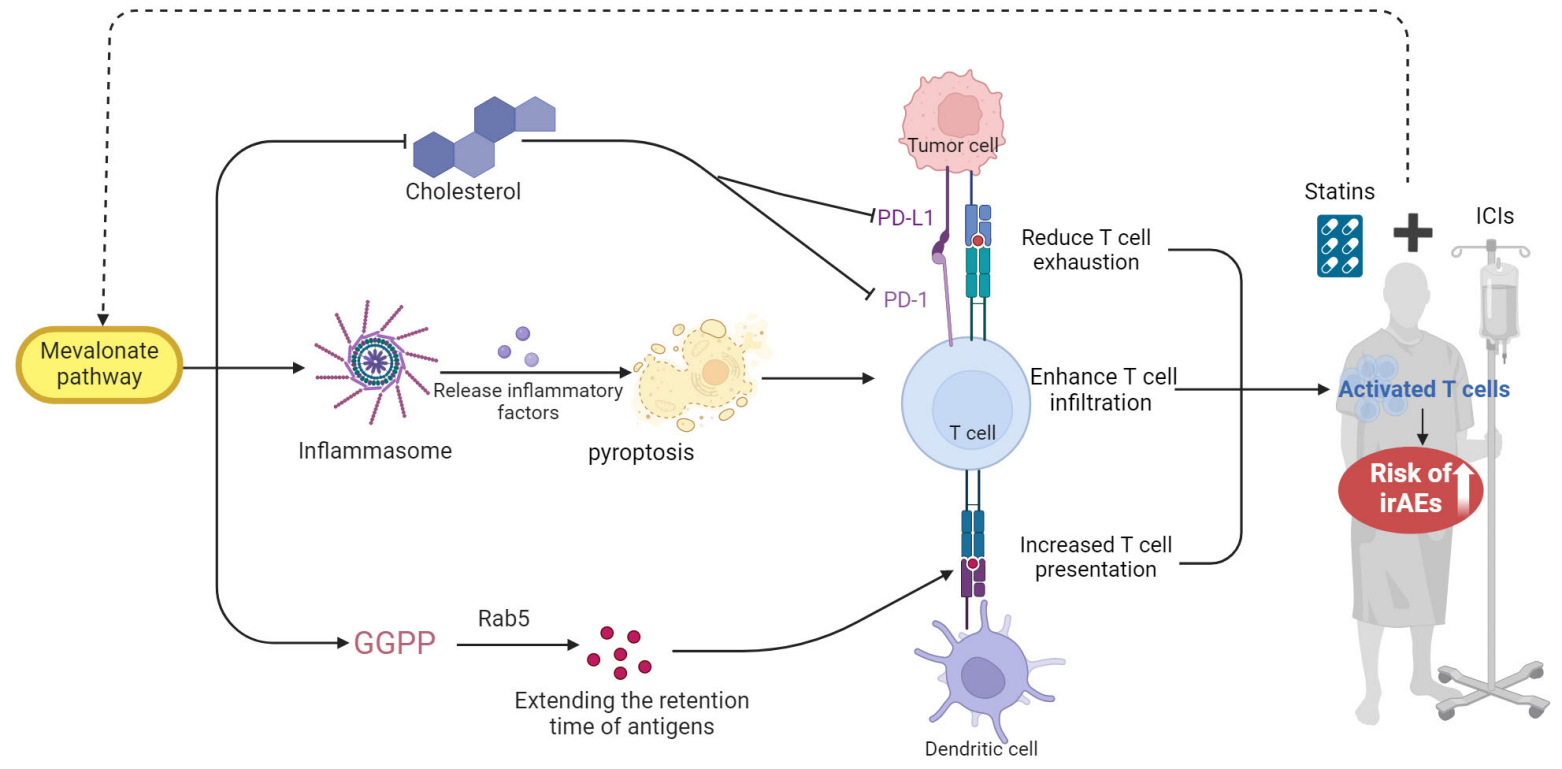
The proposed mechanism by which statin use may elevate the risk of irAEs in patients undergoing ICIs. GGPP, geranylgeranyl diphosphate. This figure was created using BioRender.com.

In summary, the heightened risk of irAEs linked to statin use may stem from excessive T-cell activation. This activation partly occurs in part by inhibiting the mevalonate pathway, which not only enhances T-cell presentation but also their infiltration. Additionally, the downregulation of inhibitory signals such as PD-1 through the cholesterol pathway can further enhance T-cell functionality. While activated T-cells may augment the anti-tumor efficacy of ICIs (30), they might also attack normal tissue cells, leading to an increase in irAEs, as shown in **Figure 10**.

Our findings indicate that individuals on statin therapy experience a higher risk of irAEs compared to non-users, with several potential mechanisms identified in the literature to explain this observation. We further sought to understand if there are differences among the seven commercially available statins. Among the most widely used statins—atorvastatin, rosuvastatin, and simvastatin—our findings relate to a higher risk of irAEs. No significant effects were observed with fluvastatin, lovastatin, pitavastatin, and pravastatin; however, due to their less frequent usage, these results may not be representative and warrant further validation with larger sample sizes. Interestingly, after propensity score matching, a lower risk of irAEs was observed in users of pravastatin. Although only 513 patients used this drug and its mechanism is not yet clearly defined, this suggests that pravastatin may be preferable for patients at heightened risk of irAEs undergoing both statin therapy and ICIs. Further studies are necessary to substantiate this hypothesis.

The differences in lipophilicity or hydrophilicity among statins may influence their pharmacokinetics and tissue selectivity. Compared to other statins, pravastatin and rosuvastatin exhibit lower lipophilicity and greater hydrophilicity. Previous research suggests that lipophilic statins, known for their non-selective diffusion into extraparenchymal tissues such as skeletal muscle, are more frequently associated with adverse drug reactions (31). However, the latest meta-analysis shows no difference in safety between lipophilic and hydrophilic statins (32). In our findings, both lipophilic and hydrophilic statins were associated with an increased risk of irAEs, showing no significant distinction between the two groups.

Interestingly, while statins are connected to an elevated risk of conditions such as anemia, arthralgia, colitis, pneumonitis, pruritus and thrombocytopenia, they appear to reduce the risk of developing Stevens-Johnson syndrome and thyroiditis. These observations suggest that the effects of statins on the immune system are complex and may vary depending on the specific immune pathways involved. Moreover, their associations with different ICI treatments also vary. Anemia, arthralgia, pneumonitis, and pruritus seem to be more strongly linked with PD-1 inhibitor regimens, while anti-PD-L1 regimens are more strongly linked with anemia, colitis, and thrombocytopenia. Hepatic impairment and muscle-related symptoms are the most commonly mentioned adverse reactions to statin use (33). Our results show that statins do not increase immune-mediated hepatitis risk, but they do increase the risk associated with the musculoskeletal system.

Comparing the toxicity profiles of PD-1, PD-L1, and CTLA-4 blockade therapies when combined with statins poses significant challenges. PD-L1 and CTLA-4 blocking antibodies are used much less frequently than PD-1 blockers, and they vary in FDA-approved indications and the types of tumors they target (34). At present, we are unable to differentiate the adverse event profiles of anti-PD-1, anti-PD-L1, and anti-CTLA-4 therapies, and our findings offer only preliminary insights and potential directions, indicating the need for more personalized investigations for the various ICIs regimens. Future research should further explore these aspects to more effectively tailor ICI therapy and statin treatment.

### 5.1 Study limitations

The primary limitations of our study stem from the reliance on the FAERS passive surveillance system, which depends on voluntary reporting. This reliance may lead to incomplete and selective reporting, resulting in data that are often inaccurate and inconsistent, thereby limiting the study’s findings and the ability to generalize results. Critically, the FAERS system lacks detailed information on patient characteristics and drug exposure, including statin dosages, durations, and other concomitant medications that may influence outcomes. Additionally, the generalizability of our findings may be limited by the diversity of the patient population, making the results not necessarily applicable to all patient groups. Lastly, while our study identified an association between statin use and an increased risk of irAEs, these findings are preliminary, and the underlying biological mechanisms are not yet fully understood. Therefore, further research is needed to elucidate these mechanisms and to validate this association through large-scale prospective studies.

## 6 Conclusions

The use of statins in patients receiving ICIs has been linked to an increased risk of irAEs, underscoring the need for careful management in this population. This association suggests a potential complex interaction between statins and the immune system, emphasizing the importance of personalized treatment strategies that address both cardiovascular and oncologic considerations. Further investigation into these underlying mechanisms is essential to optimize statin selection in patients undergoing ICI therapy.

## Data Availability

The original contributions presented in the study are included in the article/**Supplementary Material**. Further inquiries can be directed to the corresponding authors.
