# Correction: Association between statin use and immune-related adverse events in patients treated with immune checkpoint inhibitors: analysis of the FAERS database

**DOI:** 10.3389/fimmu.2025.1768895

**Published:** 2026-01-08

**Authors:** Huaju Yang, Rendong Huang, Ping Zhang, Yingtong Liu, Zheran Liu, Jiagang He, Xingchen Peng

**Affiliations:** 1 Department of Radiation Oncology, Cancer Center, West China Hospital, Sichuan University, Chengdu, Sichuan, China; 2 Hangzhou Linan Guorui Health Industry Investment Co., Ltd, Hangzhou, Zhejiang, China; 3 Department of Oncology, Chengdu Integrated TCM & Western Medicine Hospital, Chengdu First People’s Hospital, Chengdu, Sichuan, China; 4 Department of Biotherapy, Cancer Center, West China Hospital, Sichuan University, Chengdu, Sichuan, China; 5 Department of Medical Education, Kweichow Moutai Hospital, Zunyi, Guizhou, China

**Keywords:** immune checkpoint inhibitors (ICIs), statins, immune-related adverse events(irAEs), cancer, food and drug administration adverse event reporting system database (FAERS)

The funder “1.3.5 project for disciplines of excellence from West China Hospital of Sichuan University (ZYYC23006)” to “Xingchen Peng” was erroneously omitted.

The corrected **Funding** statement should read as follows:

“The author(s) declare financial support was received for the research, authorship, and/or publication of this article. The work was supported by A single center, open, randomized controlled clinical trial of Moutai Liquor regulating oral microorganisms to alleviate radiation-induced oral mucosa damage in head and neck squamous cell carcinoma (MTyk-2022-03), the National Key Research and Development Program of China (2021YFE0206600), National Natural Science Foundation of China (82172842 and 81672386), Sichuan Science and Technology Program (2022YFSY0012, 2021ZYCD011, 2021YFSY0008, 2021CDDZ-25, 2021CDZG-24), Chengdu International Science and Technology Cooperation Program (2022-GH03-00004-HZ), West China Nursing Discipline Development Special Fund Project (HXHL21008), The Post-Doctor Research Project, West China Hospital, Sichuan University (2020HXBH119), Translational Medicine Fund of West China Hospital (CGZH19002), The China Medical Board (Grant 22-482), the Clinical Research Incubation Project of West China Hospital (20HXFH037), and the 1.3.5 project for disciplines of excellence from West China Hospital of Sichuan University (ZYYC23006)”

The original version of this article has been updated.

