# Supplementary material for: Association between statin use and immune-related adverse events in patients treated with immune checkpoint inhibitors: analysis of the FAERS database
Source: Front Immunol. 2024 Oct 8;15:1439231. doi: 10.3389/fimmu.2024.1439231 (PMC11493589; doi:10.3389/fimmu.2024.1439231)
Supplement: Supplementary file 1 [file DataSheet1.docx]

Supplementary Material

## Supplementary Figures:


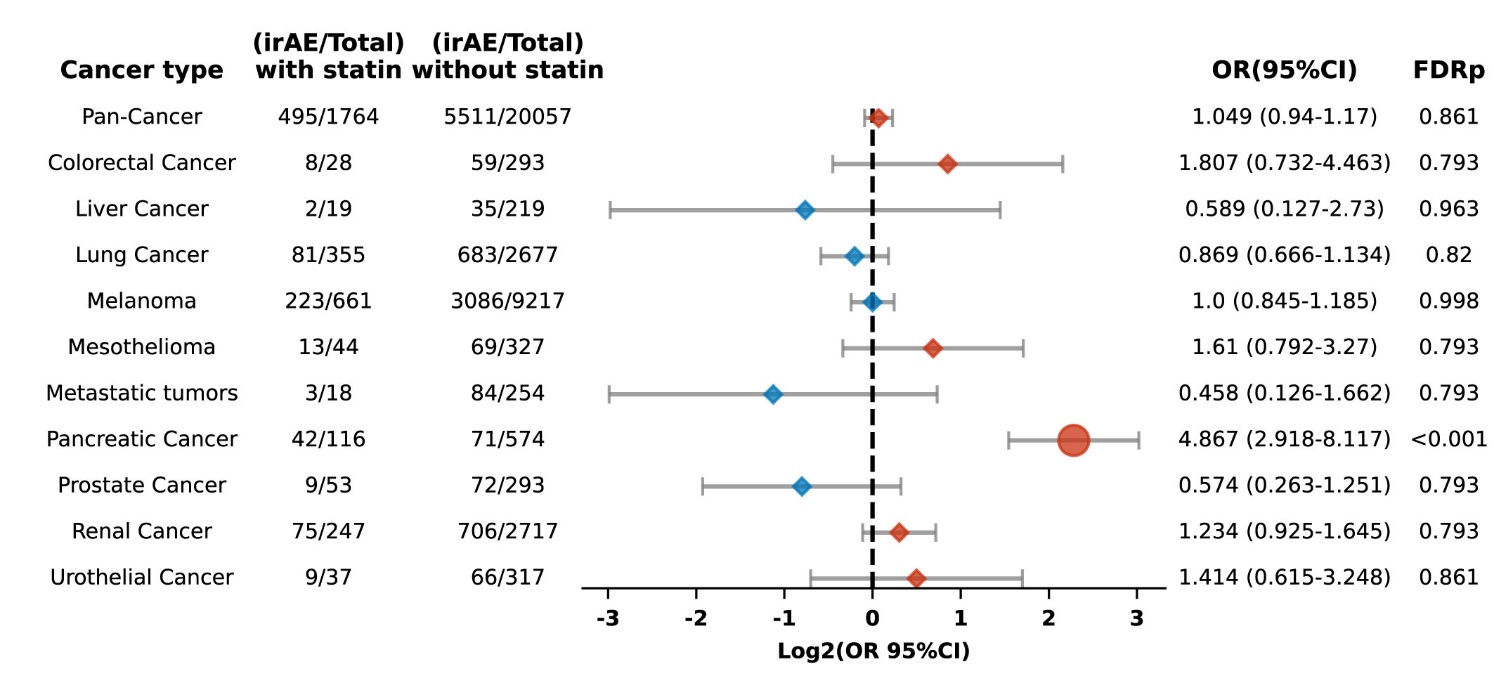


**Supplementary Figure 1.** Forest plot illustrating the relationship between statin use and irAEs across various cancer types among patients undergoing CTLA-4 blockade drugs. The icons and color coding utilized in this figure adhere to the same conventions established in Figure 1.


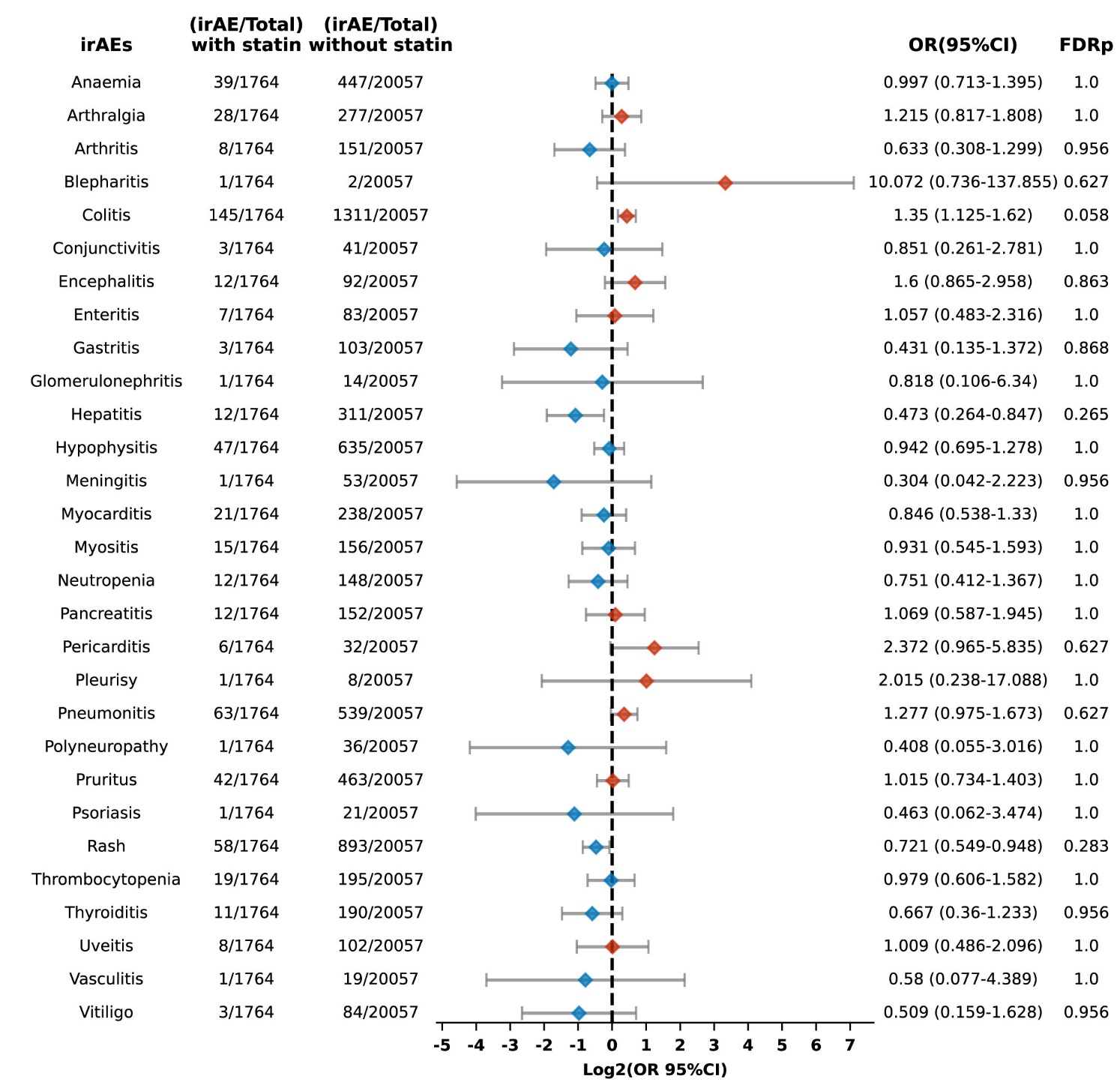


**Supplementary Figure 2.** Forest plot illustrating the connection between statin use and different irAEs among patients undergoing CTLA-4 blockade drugs. The icons and color coding utilized in this figure adhere to the same conventions established in Figure 1.
